# Supplementary figures and images for: A genome-wide association study reveals a novel regulator of ovule number and fertility in Arabidopsis thaliana
Source: PLoS Genet. 2019 Feb 11;15(2):e1007934. doi: 10.1371/journal.pgen.1007934 (PMC6386413; doi:10.1371/journal.pgen.1007934)

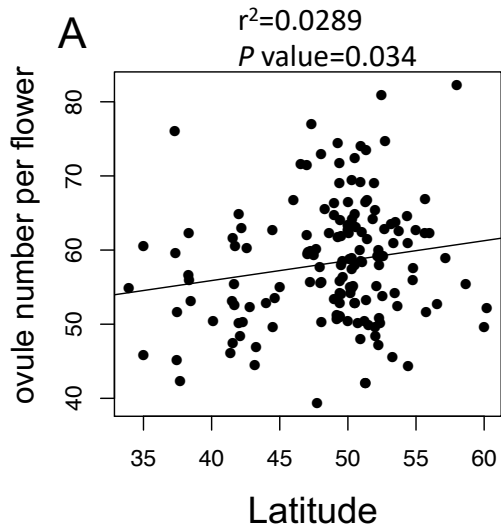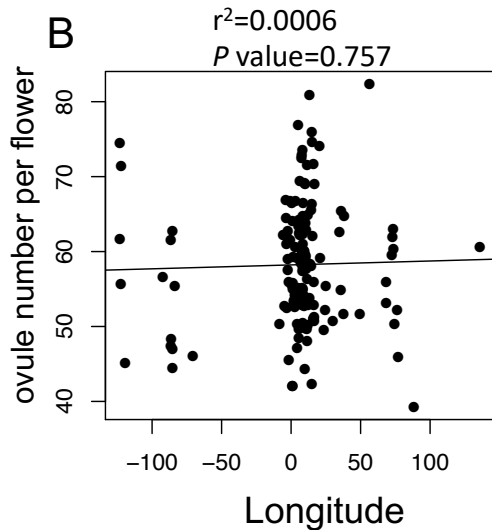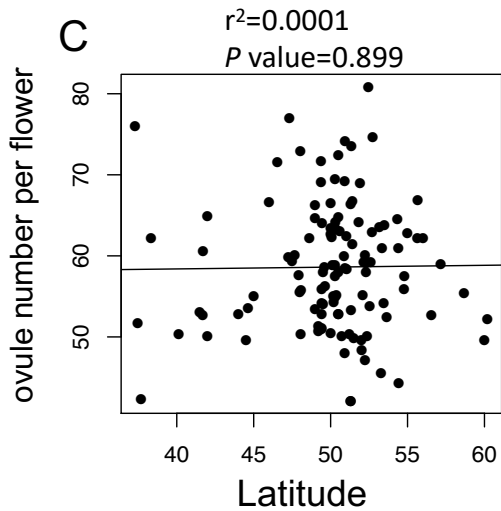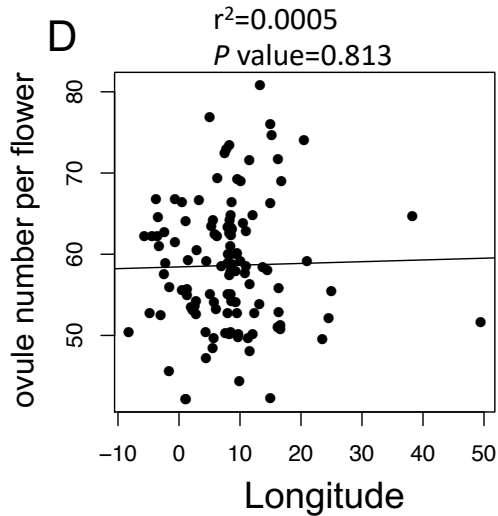

Supplement: S1 Fig — (A-B) A weakly positive correlation was detected between latitude and ovule number when all accessions were considered. (C-D) No correlation between ovule number and latitude or longitude was identified when only European accessions were tested. A Pearson correlation coefficient was computed to assess the relationship between the ovule number per flower and their geographic distribution of latitude or longitude, respectively. There was a slight correlation between the ovule number per flower and latitude distribution when accessions from all locations were tested [r2 = 0.0289, p value = 0.034]. There was no correlation between the ovule number per flower and longitude when accessions from all locations were tested [r2 = 0.0006, p value = 0.757]. There was no correlation between the ovule number per flower and latitude or longitude of the accessions’ origin when only European accessions were tested [r2 = 0.0001 or 0.0005, p value = 0.899 or 0.813], respectively. (PDF) [file pgen.1007934.s001.pdf]

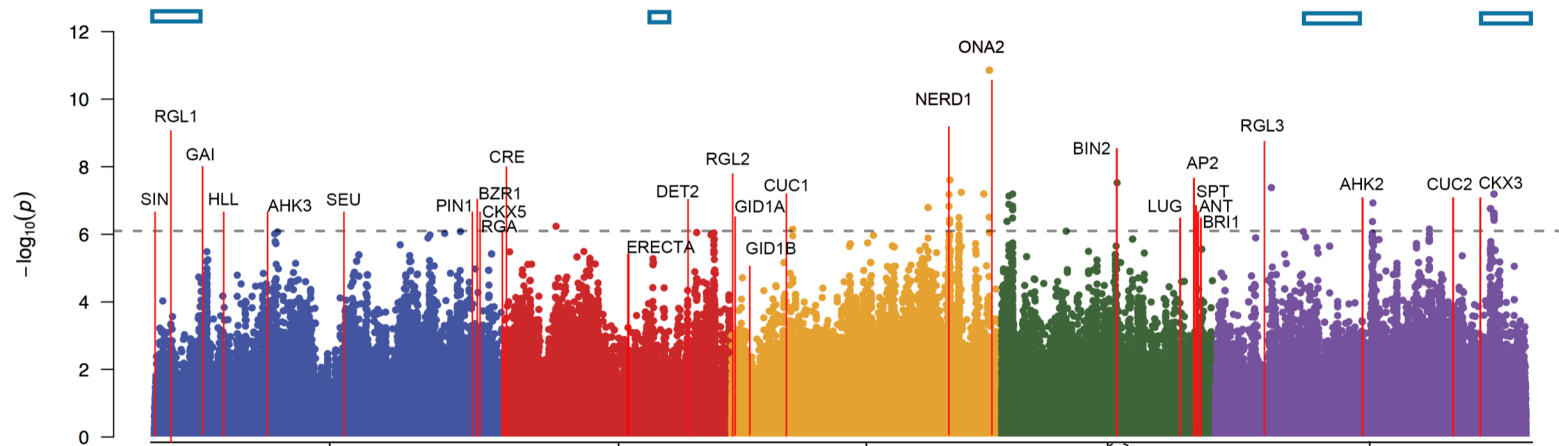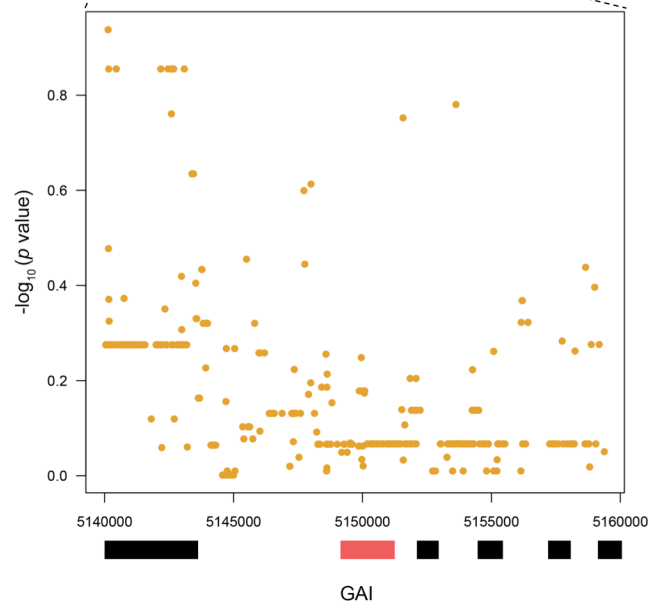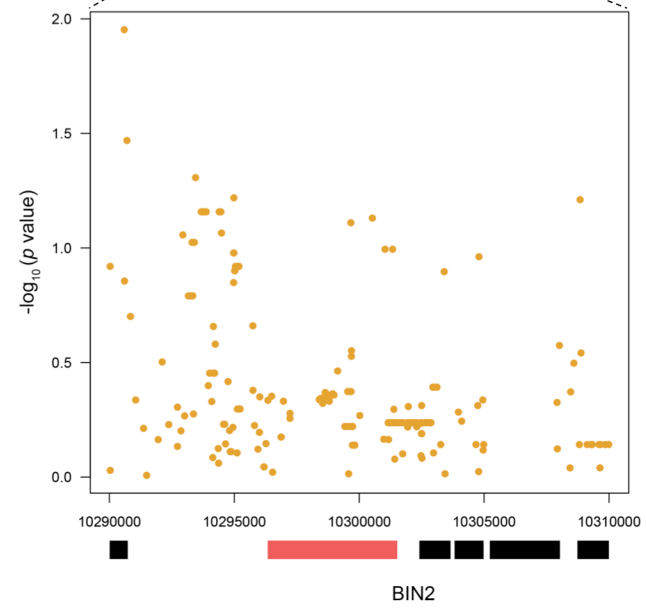

Supplement: S2 Fig — The Manhattan plot is the same as in Fig 2. Red lines indicate the known ovule number related genes. Blue boxes indicate the genomic regions underlying 4 ovule number QTL identified by [23]. (PDF) [file pgen.1007934.s002.pdf]

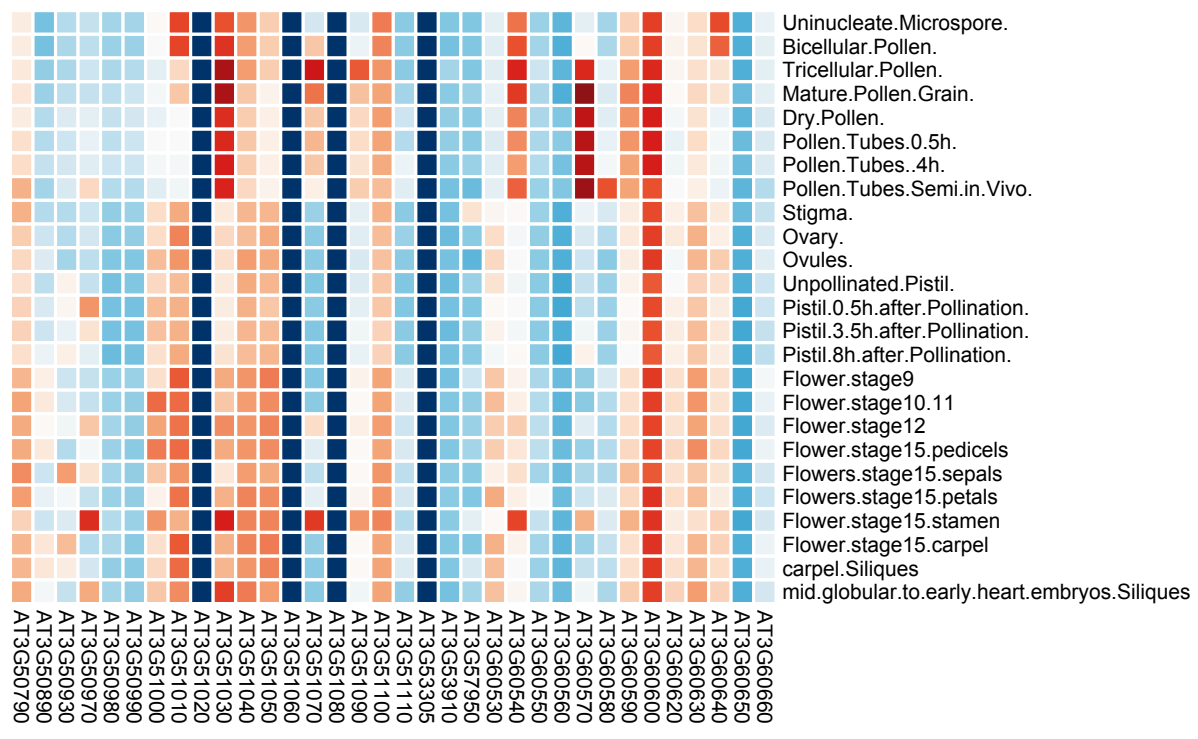

Supplement: S3 Fig — Colors from blue to red indicate the gene expression level from low to high. (PDF) [file pgen.1007934.s003.pdf]

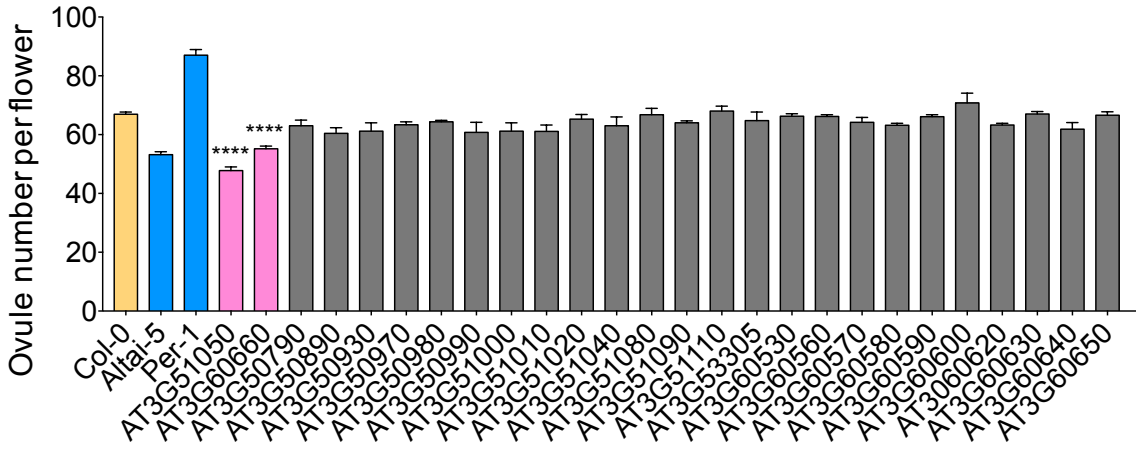

Supplement: S4 Fig — “****” indicates statistical significance (p value < 0.0001 determined by Student’s t-test). (PDF) [file pgen.1007934.s004.pdf]

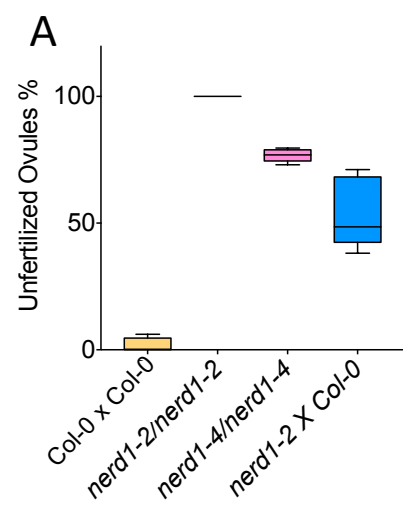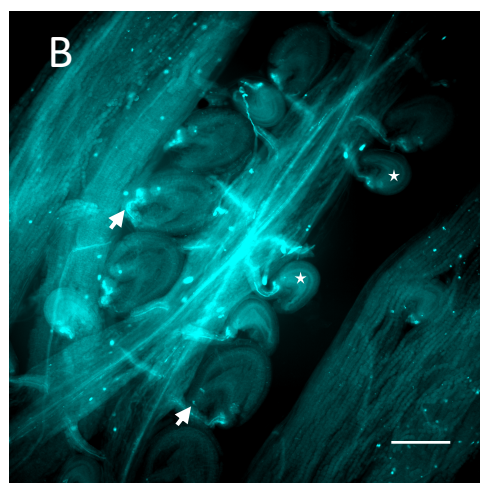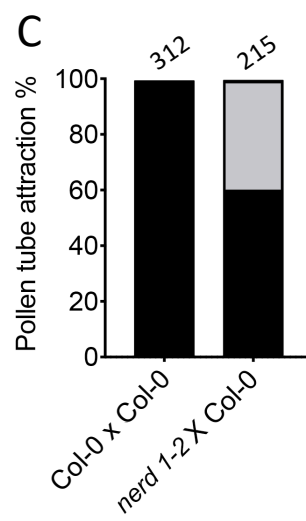

Supplement: S5 Fig — (A) Homozygous nerd1 mutants have high levels of infertility that can be partially rescued by pollinating with Col-0 wild-type pollen. (B) Aniline blue staining for nerd1-2/nerd1-2 pollinated with Col-0 pollen. Arrows indicate fertilized ovules with normal pollen tube attraction and stars indicate unfertilized ovules without pollen tube attraction. Bar = 100 μm. (C) Quantification of pollen tube attraction percentage for nerd1-2/nerd1-2 plants pollinated with Col-0 pollen (black indicates fertilized ovules with pollen tubes and gray indicates unfertilized ovules with no pollen tubes). 5 pistils were analyzed for each cross. (PDF) [file pgen.1007934.s005.pdf]

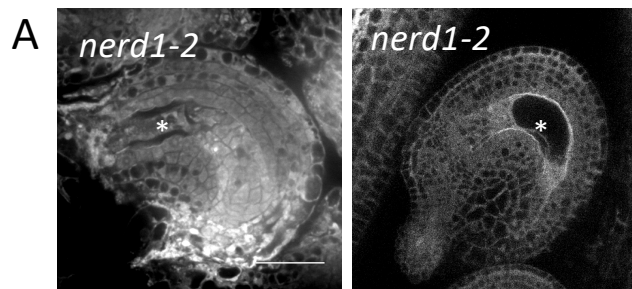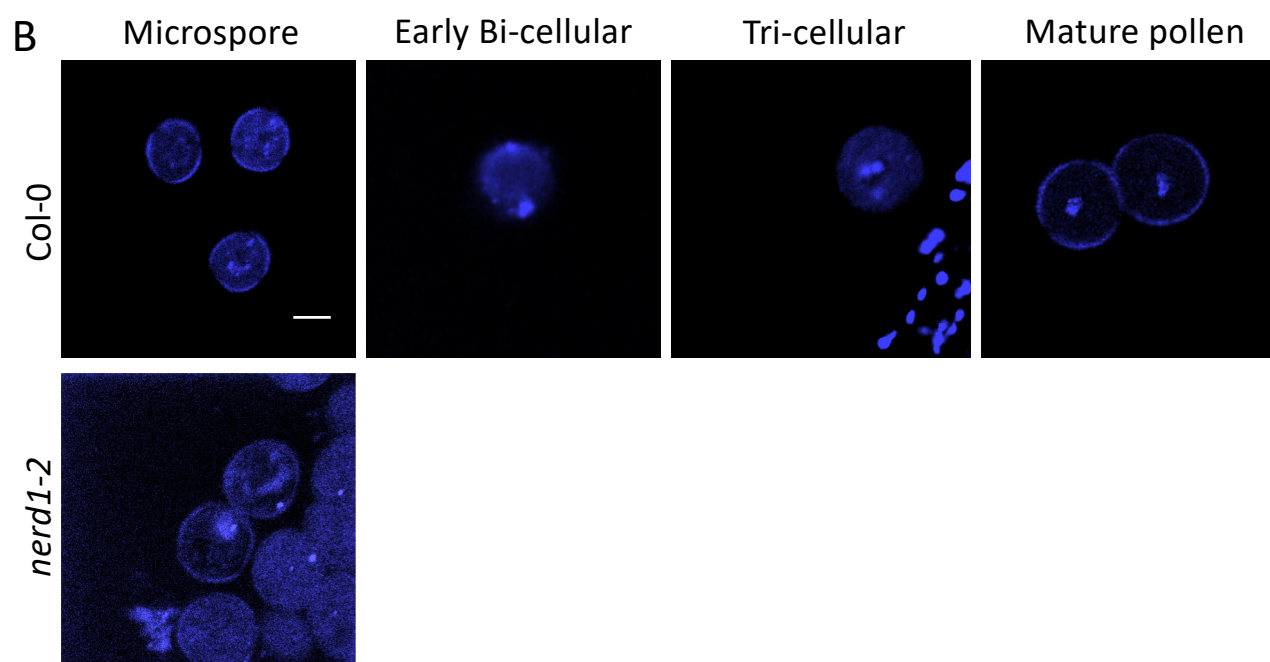

Supplement: S6 Fig — (A) Examples of defective embryo sacs in nerd1-2/nerd1-2 mature ovules. Bar = 50 μm. (B) In comparison to Col-0, nerd1-2/nerd1-2 has defective microspores and an absence of later pollen stages. Bar = 15 μm. (PDF) [file pgen.1007934.s006.pdf]

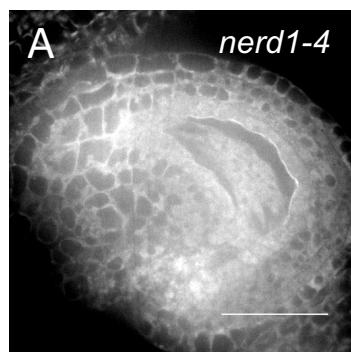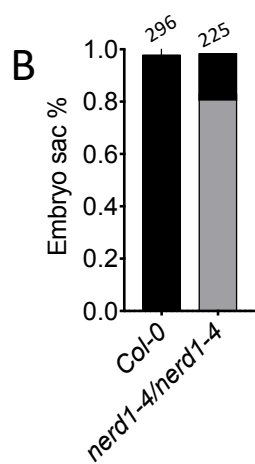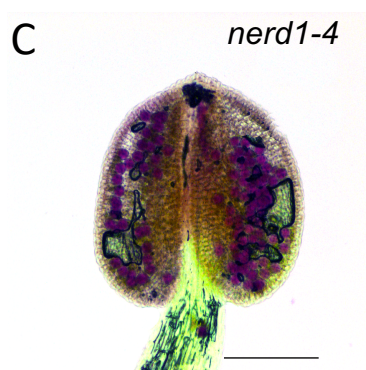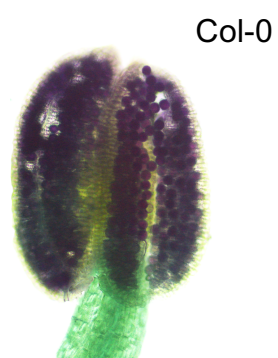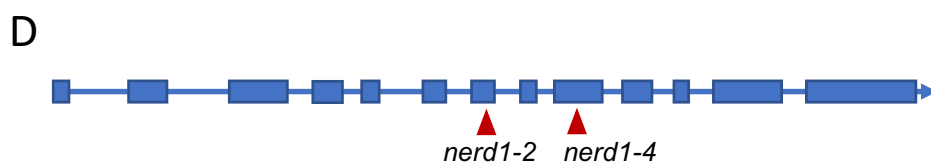

Supplement: S7 Fig — (A) Aborted embryo sac in a mature nerd1-4 ovule. Bar = 50 μm. (B) Comparison of normal (black) vs. defective (gray) embryo sac percentages in Col-0 and nerd1-4 pistils. (C) Fewer viable pollen grains are present in Alexander stained anthers of nerd1-4/nerd1-4 compared to Col-0. Bar = 100 μm. (D) T-DNA insertion sites in the NERD1 gene (boxes indicate exons and lines indicate introns). (PDF) [file pgen.1007934.s007.pdf]

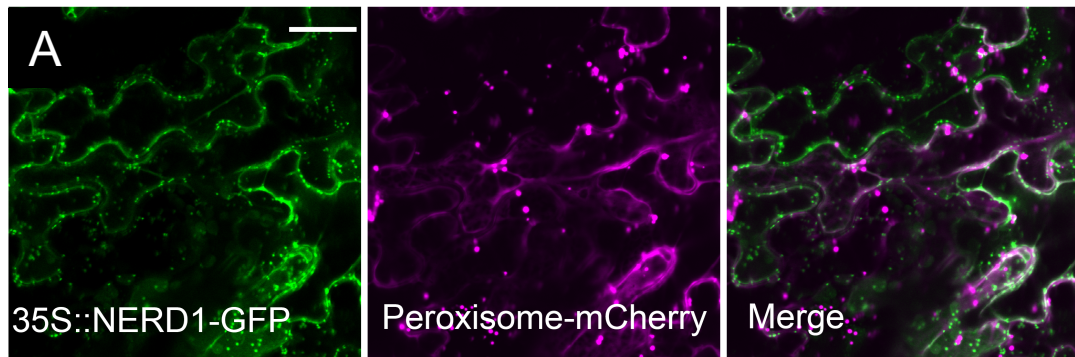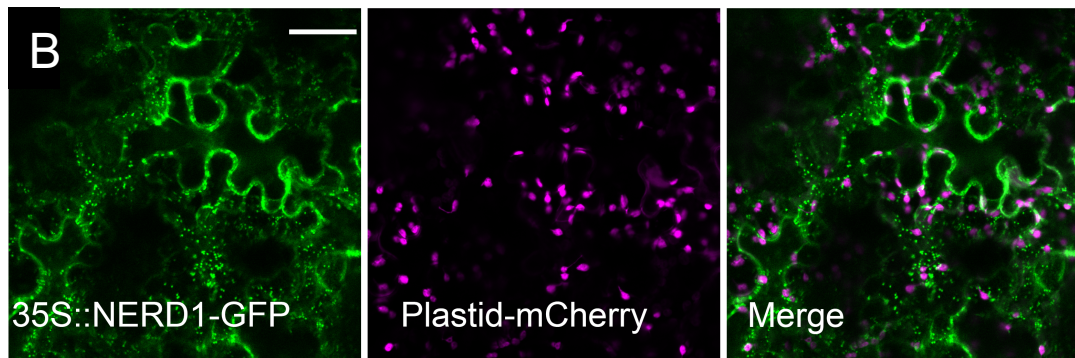

Supplement: S8 Fig — (a) 35S::NERD1-GFP (green signal) does not co-localize with Peroxisome-mCherry and (b) Plastid-mCherry (magenta) markers in N. benthamiana epidermal cells. Scale bar = 25 μm. (PDF) [file pgen.1007934.s008.pdf]

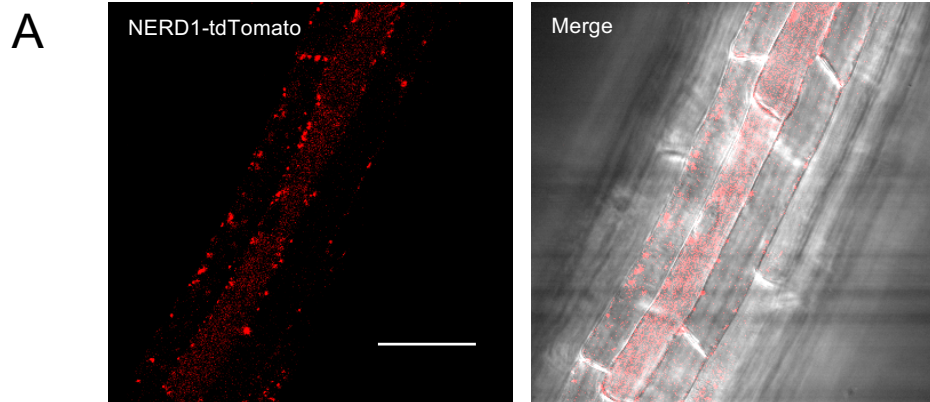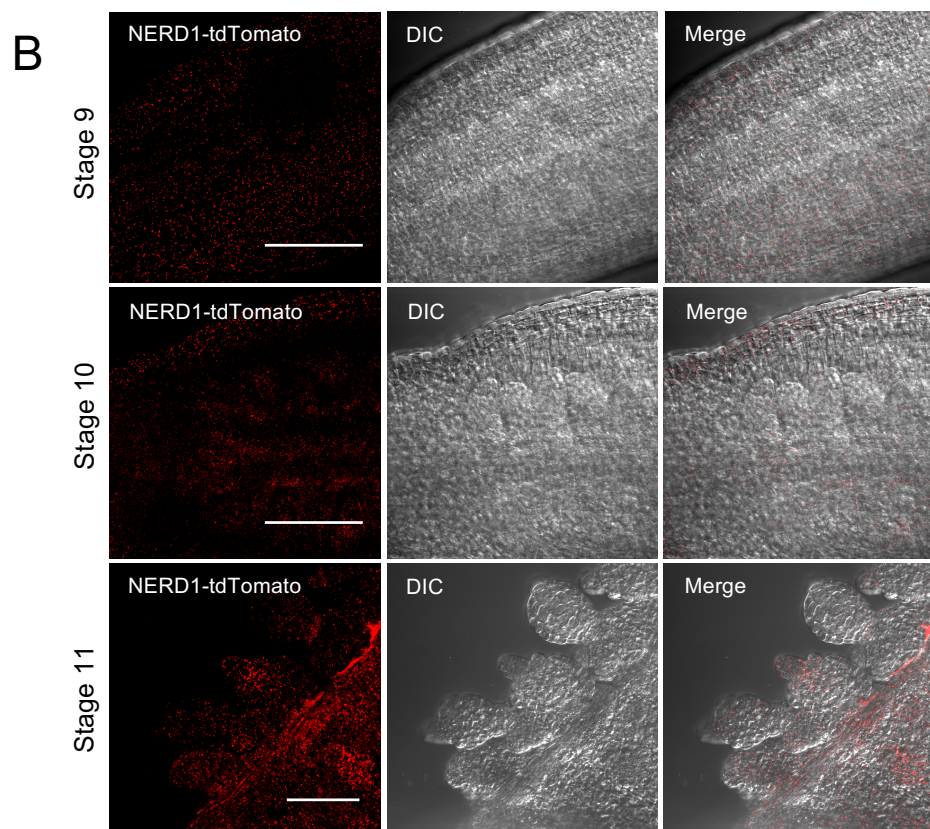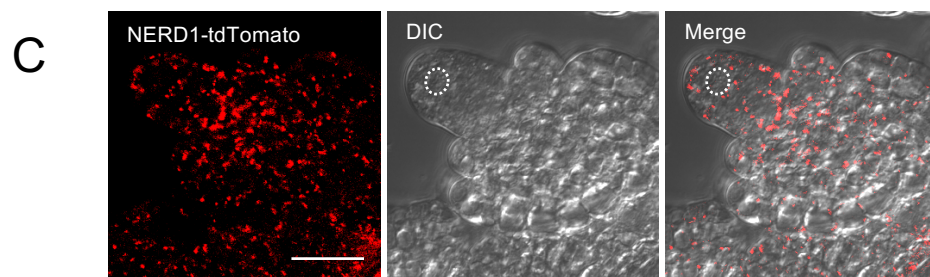

Supplement: S9 Fig — (A) NERD1-TdTomato is present in a punctate compartment in root epidermal cells. (B) NERD1 localization in ovules at flower developmental stages 9, 10 and 11. (C) Magnification of stage 11 from panel B showing punctate NERD1 accumulation in the nucellus around the megaspore mother cell (dashed circle). Bars = 30 μm (A), 20 μm (B), 10 μm (C). (PDF) [file pgen.1007934.s009.pdf]

A

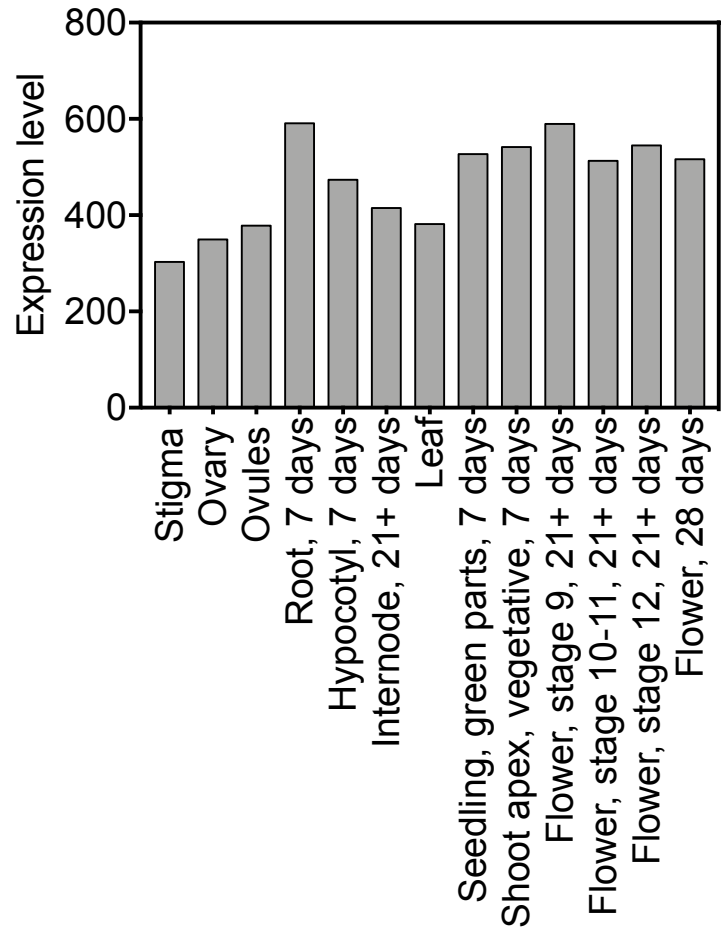

B NERD1<sub>pro</sub>::NERD1-GUS

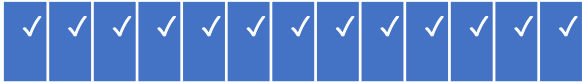

Supplement: S10 Fig — (A) NERD1 expression level in different tissues from publicly available transcriptome data in ePlant. (B) NERD1pro::gNERD1-GUS fusion construct data (see Fig 6) matches the ePlant transcriptome data. (PDF) [file pgen.1007934.s010.pdf]

A

| Accessions | Sterile plants | Normal plants |
|------------|----------------|---------------|
| Col-0      | 4              | 6             |
| Altai-5    | 1              | 9             |

B

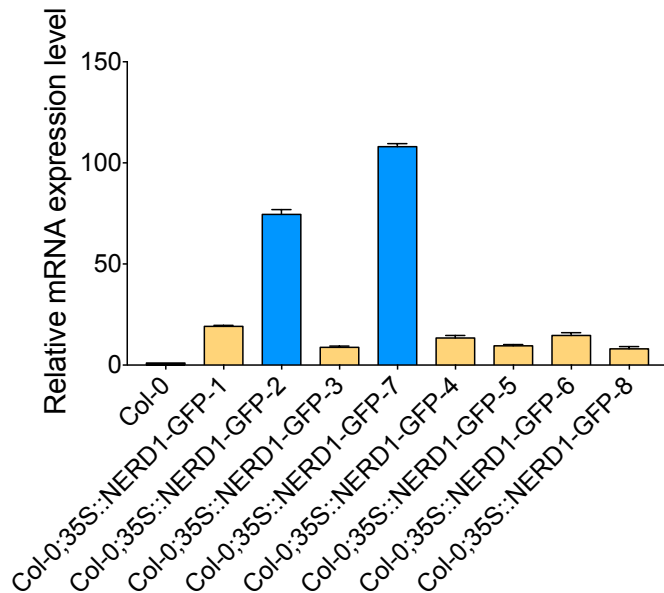

Supplement: S11 Fig — (a) The number of sterile plants and normal T1 plants in Col-0 and Altai-5. (b) qRT-PCR of NERD1 in 35S::NERD1 plants in Col-0 background. Yellow bars represent plants with normal fertility and blue bars indicate male sterile plants. (PDF) [file pgen.1007934.s011.pdf]

A

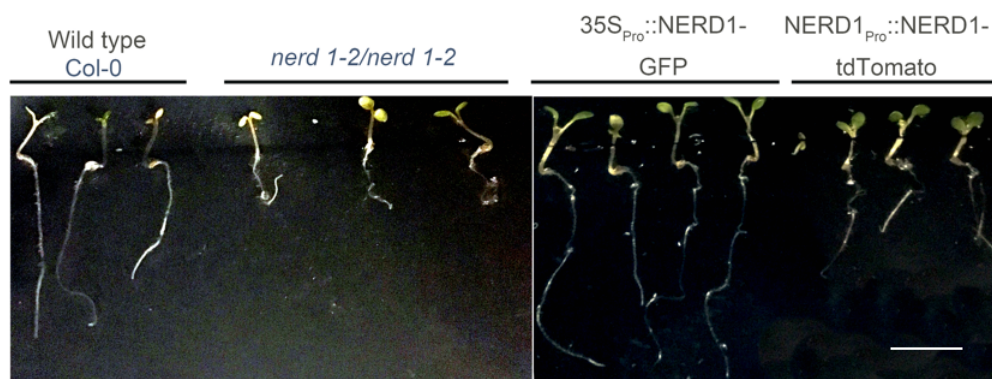

B

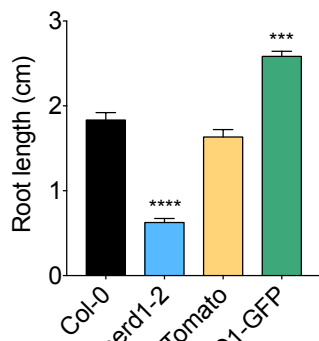

Col-0

*nerd1-2/nerd1-2*

*nerd1-2; pNERD1::NERD1-tdTomato*

*Col-0; 35S::NERD1-GFP*

Supplement: S12 Fig — (a) Root phenotype in Col-0, nerd1-2/nerd1-2, 35S::NERD1, and the NERD1 complementation line. nerd1 mutants have significantly shorter roots while overexpression of NERD1 leads to longer roots than the Col-0 control. Bar = 0.7 cm. (b) Bar graph of the quantification of the root length. “****” indicates statistical significance (p value < 0.0001 determined by Student’s t-test). “***” indicates statistical significance (p value < 0.001 determined by Student’s t-test, n = at least 9 individuals for each genotype). (PDF) [file pgen.1007934.s012.pdf]

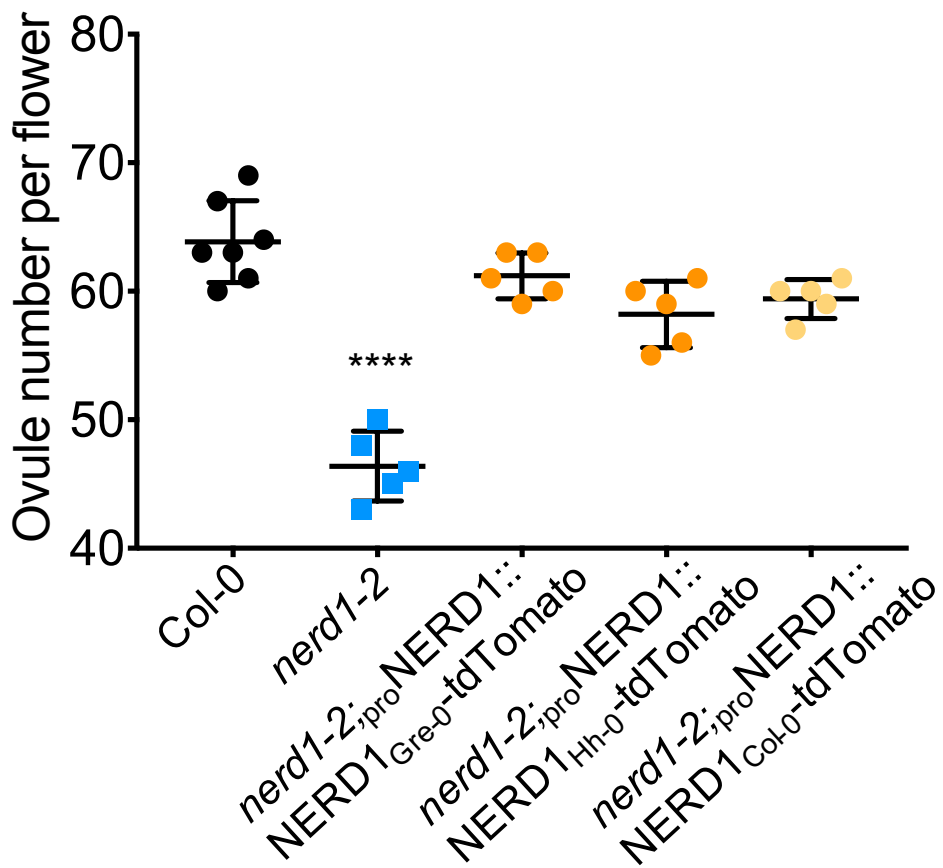

Supplement: S13 Fig — (p value < 0.0001 determined by Student’s t-test). (PDF) [file pgen.1007934.s013.pdf]
